# Systemic exosomal miR-193b-3p delivery attenuates neuroinflammation in early brain injury after subarachnoid hemorrhage in mice

**DOI:** 10.1186/s12974-020-01745-0

**Published:** 2020-02-25

**Authors:** Niansheng Lai, Degang Wu, Tianyu Liang, Pengjie Pan, Guiqiang Yuan, Xiang Li, Haiying Li, Haitao Shen, Zhong Wang, Gang Chen

**Affiliations:** 1 Department of Neurosurgery & Brain and Nerve Research Laboratory, The First Affiliated Hospital of Soochow University, 188 Shizi Street, Suzhou, 215006 Jiangsu Province China; 2 Department of Neurosurgery, First Affiliated Hospital of Wannan Medical College, 2 West Zheshan Road, Wuhu, Anhui Province China; 3 Non-coding RNA Research Center of Wannan Medical College, Wuhu, Anhui Province China

**Keywords:** Subarachnoid hemorrhage, Neuroinflammation, Exosomes, Brain delivery

## Abstract

**Background:**

Inflammation is a potential crucial factor in the pathogenesis of subarachnoid hemorrhage (SAH). Circulating microRNAs (miRNAs) are involved in the regulation of diverse aspects of neuronal dysfunction. The therapeutic potential of miRNAs has been demonstrated in several CNS disorders and is thought to involve modulation of neuroinflammation. Here, we found that peripherally injected modified exosomes (Exos) delivered miRNAs to the brains of mice with SAH and that the potential mechanism was regulated by regulation of neuroinflammation.

**Methods:**

Next-generation sequencing (NGS) and qRT-PCR were used to define the global miRNA profile of plasma exosomes in aSAH patients and healthy controls. We peripherally injected RVG/Exos/miR-193b-3p to achieve delivery of miR-193b-3p to the brain of mice with SAH. The effects of miR-193b-3p on SAH were assayed using a neurological score, brain water content, blood-brain barrier (BBB) injury, and Fluoro-Jade C (FJC) staining. Western blotting analysis, enzyme-linked immunosorbent assay (ELISA), and qRT-PCR were used to measure various proteins and mRNA levels.

**Results:**

NGS and qRT-PCR revealed that four circulating exosomal miRNAs were differentially expressed. RVG/Exos exhibited improved targeting to the brains of SAH mice. MiR-193b-3p suppressed the expression and activity of HDAC3, upregulating the acetylation of NF-κB p65. Finally, miR-193b-3p treatment mitigated the neurological behavioral impairment, brain edema, BBB injury, and neurodegeneration induced by SAH, and reduced inflammatory cytokine expression in the brains of mice after SAH.

**Conclusions:**

Exos/miR-193b-3p treatment attenuated the inflammatory response by acetylation of the NF-κB p65 via suppressed expression and activity of HDAC3. These effects alleviated neurobehavioral impairments and neuroinflammation following SAH.

## Background

Aneurysmal subarachnoid hemorrhage (SAH) typically results from a ruptured aneurysm. It is a clinical syndrome associated with mortality of 45% and morbidity of approximately 6–16 per 100,000 individuals annually worldwide, often in young individuals. SAH accounts for 5–7% of the total incidence of stroke [1, 2]. A major prognostic determinant is the character of the initial hemorrhage that causes early brain injury (EBI) and early cerebral vasospasms (CVS) and may be associated with delayed cerebral ischemia (DCI) [3]. Several lines of evidence suggest that EBI after SAH may be the leading factor contributing to unfavorable outcomes following SAH [4, 5]. The evidence suggests that several pathophysiological disorders during EBI activate inflammatory cascades [6]. It is of paramount importance to develop novel neuroprotective therapies to enhance recovery in the period of EBI after SAH.

MicroRNAs (miRNAs) are a family of non-coding RNAs of 17–24 nucleotides that regulate the expression of several target genes at the post-transcriptional level [7–11]. Previous studies demonstrated that miRNAs were involved in numerous physiological/pathological processes and were particularly appealing diagnostic and therapeutic tools for various diseases, including stroke, Parkinson’s disease, traumatic brain injury (TBI), and Alzheimer’s disease. Despite the fact that expression levels of miRNAs have measured in cerebrospinal fluid and in circulation, there are few studies investigating changes of miRNAs after SAH [12–14]. The blood-brain barrier (BBB) has been proven to be a major obstacle for delivery of drugs to the brain. It has been estimated that 98% of all potent molecules have no clinical effect because of their inability to cross the BBB [11, 15].

Several lines of evidence suggested that exosomes, which are lipid membrane vesicles of 30 to 100 nm in diameter, cross the BBB and specialize in long-distance intercellular communication, facilitating transfer of proteins, lipids, functional mRNAs, and microRNAs for subsequent protein expression in target cells [16]. Exosomes released by brain cells are able to cross the BBB and can be detected in the systemic circulation. Similarly, endothelial and peripheral cells secrete exosomes into the circulation. Exosomes can be enriched from peripheral blood samples and can be used to detect various proteins, lipids, and nucleic acids [17]. Exosomes have been used as drug delivery vehicles for the treatment of several CNS diseases [11, 18–21]. Exosomes are currently under investigation as therapeutic options for stroke [22]. Rabies virus glycoprotein (RVG) has been engineered for the exosomal surface using fused protein lysosome-associated membrane glycoprotein 2b (Lamp2b)-RVG, a preferred option to address this problem, because they cross the BBB and transport miRNAs specifically into the CNS [11, 23]. Unlike intracerebroventricular injections, RVG/exosomes (RVG/Exos) are injected intravenously and are thereby easily used for noninvasive treatment of CNS diseases. They are capable of transporting small molecules and macromolecular drugs rapidly to the CNS within the liver or degradation by mononuclear phagocyte system.

To the best of our knowledge, our study is the first of exosome-based miRNA therapy in SAH. We measured expression profiles of plasma exosomal miRNAs in patients with SAH using exosomal miRNA sequencing and qRT-PCR. Then, we tested whether exosomes derived from bone marrow mesenchymal stem cells (BMSCs) would transfer differentially expressed plasma exosomal miRNAs to brain tissues, leading to upregulation of miRNAs in brain tissues in mice after SAH. We evaluated the possibility that Exos/miRNAs inhibited neuroinflammation and exhibited neuroprotective effects against EBI in SAH.

## Methods

### Ethics and animals

Study participants were recruited from the Department of Neurosurgery at The First Affiliated Hospital of Wannan Medical College, Wuhu, China. The study was performed in accordance with the Declaration of Helsinki. Written informed consent was received from participants or valid proxies (family or a professional not directly involved in the study). All experiments were approved by the Ethics Committee of the First Affiliated Hospital of Soochow University and Research Ethics Committee of Wannan Medical College, and were performed according to the guidelines of the National Institutes of Health on the care and use of animals. All adult male C57BL/6 mice weighing 25–30 g were purchased from the Animal Center of the Chinese Academy of Sciences, Shanghai, China. All mice were housed in temperature- and humidity-controlled animal quarters with a 12-h light/dark cycle. Every effort was made to minimize the number of animals used and their suffering.

### SAH model in mice

As described in a previous study [24], mice were intraperitoneally anesthetized with 4% chloral hydrate (0.4 g/kg body weight). They were then placed in the stereotaxic apparatus. A specially made needle with a rounded tip and a side hole was stereotactically inserted into the suprachiasmatic cistern to produce the SAH model. The needle (3.5 mm anterior to the bregma, angle of 28–30°) was inserted in the midline until it reaches the skull (2 mm anterior to the chiasma, 0.5 mm retracted). Next, 0.1 ml non-heparinized allologous arterial blood was slowly injected into the suprachiasmatic cistern over 20 s; 0.1 ml saline was injected for the sham group. To prevent dehydration, the mice were injected subcutaneously with 1 ml of 0.9% saline after surgery. We observed that the inferior basal temporal lobe was always stained with blood. Plasma and brain tissues surrounding the hemorrhage located in the temporal base were sampled and subjected to analysis after the mice were sacrificed at the indicated time points.

### Cell culture

Bone marrow from 6-week-old adult male mice was mechanically harvested from femurs as previously described [25]. Cells were washed in PBS and suspended in DMEM supplemented with 20% fetal bovine serum and antibiotics (all from GIBCO, USA). Three days later, non-adherent cells were removed by replacing fresh medium, and cells remaining tightly adhered to the plastic flasks were considered to be *P0* BMSCs. BMSCs were used until the eighth passage (*P8*) for exosome collection.

### Lentivirus production and infection

The 1356-bp sequence of the exosomal membrane protein gene Lamp2b (lysosomal-associated membrane protein 2b) was fused with the targeting peptide RVG and was amplified using 293T cell cDNA as a template, with the following primers: m-RVG-lamp2b-F and m-RVG-lamp2b-R (Table 1). EcoR I and BamH I are endonuclease sites for plasmid construction with the pHBLV-CMV-MCS-3FLAG-EF1- ZsGreen-T2A-PURO vector. The sequences of all constructs were confirmed using DNA sequencing (HanBio Biotech, China). The cells (2 × 10^5^ per well in 6-well plates) were transfected with LipoFiter™ Liposomal Transfection Reagent (Hanbio Biotechnology, China), as per the protocol provided by the lentivirus manufacturer. Retroviral infection was performed as previously described [26].

**Table 1:** Real-time PCR primers used for quantification of mRNA expression in this study

Primer name	Sequence (5′ → 3′)	
m-RVG-lamp2b	Forward	CACCATTTGGATGCCCGAGA
	Reverse	CCGTTGGATGCTCTCTTCCC
*Hdac1*	Forward	ACGGCATTGACGACGAATCCTATG
	Reverse	CTGAGCCACACTGTAAGACCACTG
*Hdac2*	Forward	AGACTGGAATAGGACGGACGGATG
	Reverse	GTCATTTACCCAAGGGCTGGCTAC
*Hdac3*	Forward	TGGAACAGGTGACATGTATGAA
	Reverse	GAAAAGGTGCTTGTAACTCTGG
*Hdac8*	Forward	GATACTATTGCCGGAGATCCAA
	Reverse	GATAGCGTTTTCCCTAGGATGA
*Rela*	Forward	GCTACACAGGACCAGGAACAGTTC
	Reverse	CTTGCTCCAGGTCTCGCTTCTTC
*BCL-2*	Forward	GATGACTTCTCTCGTCGCTAC
	Reverse	GAACTCAAAGAAGGCCACAATC
*Caspase-3*	Forward	GAAACTCTTCATCATTCAGGCC
	Reverse	GCGAGTGAGAATGTGCATAAAT
*IL-1β*	Forward	GCAGAGCACAAGCCTGTCTTCC
	Reverse	ACCTGTCTTGGCCGAGGACTAAG
*IL-6*	Forward	ACGTAGCTAGCTAGTCGGTATG
	Reverse	TCGTAGCTTGGCTAGTCGATCG
*TNF-α*	Forward	ATGTCTCAGCCTCTTCTCATTC
	Reverse	GCTTGTCACTCGAATTTTGAGA
*β-actin*	Forward	GCTGTCCCTGTATGCCTCTG
	Reverse	CGCTCGTTGCCAATAGTGATG
MiRNA-193b-3p	Forward	AACTGGCCCTCAAAGTCCCGCT
MiRNA-486-3p	Forward	gCGGGGCAGCTCAGTACAGGAT
MiRNA-369-3p	Forward	accggccgcggAATAATACATGGTTGATCTTTT
MiRNA-410-3p	Forward	ccgcgggAATATAACACAGATGGCCTGT
MiRNA-136-3p	Forward	ccgcggCATCATCGTCTCAAATGAGTCT
MiRNA-195-5p	Forward	cgccggTAGCAGCACAGAAATATTGGC
Cel-miR-39	Forward	cgUCACCGGGUGUAAAUCAGCUUG
Universal reverse primers	Reverse	CAGGTCCAGTTTTTTTTTTTTTTTCGT

### Isolation and characterization of exosomes

Exosomes were purified from cell culture supernatants in serum-free medium of BMSCs. Prior to culture medium collection, BMSCs were washed twice with PBS and were cultured in serum-free medium at 37 °C in a humidified atmosphere of 5% CO_2_ for 48 h. The culture medium supernatants or plasma samples were collected and subjected to sequential ultracentrifugation at 2000*g* for 10 min at 4 °C to remove cell debris and then passed through 0.22 μm filters, spun at 10,000*g* for 30 min, then 100,000*g* for 4 h at 4 °C. The exosomes were washed once with PBS and resuspended for further characterization.

Exosomes were adsorbed on carbon-coated nickel grids for 1 h, subsequently washed three times with PBS for 5 min, and fixed with 2% formaldehyde for 10 min. Samples were contrasted using uranyl acetate and lead citrate (Sigma-Aldrich, USA). After three washings in deionized water, grids were dried for several minutes and finally examined using a TECNAI-10 transmission electron microscope (TEM; Philips, Netherlands).

### MiR-193b-3p loading

Exosomes at total protein concentration of 20 μg (measured using a BCA Assay kit, Beyotime) and 20 μl of miR-193b-3p mimics or scrambled miRNAs (GenePharma, China) were mixed in 180 μl of nucleofector buffer (Cell Line Nucleofector Kit V, Amaxa) and electroporated at 350 V and 150 μF in Nucleofector IIs/2b device. To remove the unincorporated miRNA mimics, exosomes were washed in cold PBS (4 °C) twice by sequential ultracentrifugation. Efficiency of transfection was validated using qRT-PCR for detection of miR-193b-3p levels.

### RNA isolation and qRT-PCR

Total RNA was extracted from exosomes or brain tissues using QIAzol Lysis Reagent (Qiagen, Germany) according to the manufacturer’s instructions.

For analysis of mRNA levels, reverse transcription (RT) was performed using a Fast Quant RT Kit (with gDNase; Tiangen Biotech, China), and cDNAs were used for qRT-PCR using SuperReal PreMix Color (SYBR Green; Tiangen Biotech, China). The primer sequences are listed in Table 1.

For analysis of miRNA levels, total RNA was reverse-transcribed to cDNA using an miRcute miRNA First-Strand cDNA Synthesis Kit (Tiangen Biotech, China), and qRT-PCR was carried out using an miRcute miRNA qPCR Detection Kit (SYBR Green; Tiangen, Biotech, China).

All PCR reactions were run in triplicate, and mRNA or miRNA levels were expressed relative to levels of β-actin, cel-miR-39, or U6 snRNA. The results of PCR reactions of mRNA and miRNA expression in tissues and medium were calculated using the 2^−ΔΔCt^ method, and miRNA expression in plasma was calculated using the 2^−ΔCt^ method.

### Western blot analysis

As described previously [5], frozen samples or BMSCs cells were lysed mechanically in lysis buffer (Beyotime, China). The bicinchoninic acid (BCA) method was used to measure the concentration of brain tissues using an enhanced BCA Protein Assay kit (Beyotime, China). Molecular weight markers (5 μl/lane; Thermos Scientific, USA) and protein samples (20 μg/lane) were separated on 10% sodium dodecyl sulfate-polyacrylamide gels (SDS-PAGE) and electrophoretically transferred onto polyvinylidene difluoride membranes (PVDF, Millipore Corporation, USA). Then, membranes were blocked with 5% non-fat milk for 1 h at room temperature and then were incubated with primary antibodies in 5% BSA (diluted in PBS with 0.1% Tween 20) overnight at 4 °C. The primary antibodies were as follows: goat anti-Lamp2b (1:1000, Abcam, USA), mouse anti-β-actin (1:5000, Abcam, USA), mouse anti-CD63 (1:1000, Abcam, USA), rabbit anti-Alix (1:1000, Abcam, USA), rabbit anti-GM130 (1:1000, Abcam, USA), rabbit anti-HDAC3 (Histone deacetylase 3) (1:5000, Abcam, USA), rabbit anti-acetyl-NF-κB p65 (1:1000, CST, USA), and mouse anti-GAPDH (1:5000, Abcam, USA). Corresponding HRP-conjugated anti-goat, anti-rabbit, or anti-mouse (1:10,000, CST, USA) secondary antibodies were incubated for 2 h at room temperature. Bands were visualized using an enhanced chemiluminescence (ECL) kit (Affinity, China). The relative quantity of proteins was analyzed using Image J software (NIH, USA) and normalized to quantities of loading controls.

### Enzyme-linked immunosorbent assay

At 24 h post-SAH, mice were anesthetized and brain tissues were removed. The brain tissues were mechanically homogenized in 0.9% normal saline at 200 mg/ml and centrifuged at 12,000 rpm for 10 min at 4 °C. The concentrations of IL-1β, IL-6, and TNF-α in brain tissue homogenates were quantified using specific enzyme-linked immunosorbent assay (ELISA) kits for mice (Elabscience Biotechnology, China) according to the manufacturer’s instructions. The final concentration of cytokines was measured using OD values.

### Neurological behavioral impairment

At 24 h after SAH, all mice were examined for neurological behavioral impairment by an independent investigator blinded to procedure information using the Garcia test [27]. The Garcia test consists of spontaneous activity, tail suspension movement of all limbs, forelimb outstretching, climbing, touching of trunk, and vibrissae touching. Every test was scored as 0 to 3. An 18-point scoring system was used to evaluate the sensorimotor deficits. Higher scores represent milder neurological deficits [6, 27].

### Brain edema

At 24 h post-SAH, mice were anesthetized and brain tissues were removed. Samples removed from brainstem and cerebellum were weighed immediately (wet weight, WW), and then dried for 72 h at 100 °C to obtain dry weight (DW). The percentage of brain water content (BWC) was calculated as: [(wet weight − dry weight)/wet weight] × 100%.

### Fluoro-Jade C staining

Neurodegeneration was detected using Fluoro-Jade C (FJC) staining as previously described [6]. After deparaffinization and rehydration, the brain sections were successively incubated with 80% alcohol containing 1% NaOH for 5 min, 70% alcohol for 2 min, 0.06% potassium permanganate for 10 min, and 0.0001% FJC (AG325, Millipore, Germany) working solution for 30 min. Next, sections were washed and dried in an incubator for 10 min and cleared in xylene and coverslipped with a non-aqueous, low-fluorescence, styrene-based mounting medium (Sigma-Aldrich, USA). Microscopy of the stained brain sections was performed by an experienced pathologist who was blinded to the experimental condition.

### Blood-brain barrier injury

BBB permeability was described in a previous report [28]. Briefly, we injected mice with 2% Evan’s blue dye (5 ml/kg, Sigma-Aldrich) at 24 h post-SAH. One hour later, the mice were subjected to systemic intracardiac perfusion with PBS to clear intravascular Evan’s blue dye. The mice were euthanized, with brain samples subsequently harvested and homogenized in 50% trichloroacetic acid. Evan’s blue dye in supernatants was measured at 620 nm using a spectrofluorophotometer. The results were expressed as micrograms of Evan’s blue per gram of brain tissue.

### Statistical analysis

All data were expressed as mean ± standard deviation. Before analysis, data sets in each group were tested for normality of distribution using the Kolmogorov-Smirnov test. Differences between groups were assessed using the Mann-Whitney *U* test and/or the two-sided unpaired Student’s test. Multiple comparisons between more than two groups were made using the Kruskal-Wallis test or ANOVA. Differences were considered statistically significant when *p* < 0.05. MedCalc version 13.0.0 (Broekstraat 529030, Mariakerke, Belgium) was used for all statistical analyses.

## Results

### Distinct plasma exosomal miRNA profiles in SAH patients

MiRNA sequence expression of six plasma samples from three control individuals (two women and one man, ages 58, 64, and 60, respectively) and three SAH patients (two women and one man, ages 55, 63, and 66, respectively) was analyzed to identify potential biomarkers. Plasma was taken at 24 h after SAH and was obtained in the fasting state in each healthy control. Next-generation sequencing (NGS) of these samples yielded 9 to 15 million reads aligned to the reference human genome sequence. These reads corresponded to > 50,000 different RNA sequences. From a list of 2139 known miRNAs, only 746 were considered as being expressed, with raw read counts ≥ 1 in at least one sample. When we analyzed the expression spectrum of individual miRNAs in SAH patients and control plasma exosome samples, we found distinct profiles of expression (Supplementary data). To further assess the differences between the groups, we performed global statistical analysis to detect truly differentially expressed miRNA sequences (adjusted *p* value < 0.05, |log2 (fold change| > 1 and expression level not low)). NGS permitted identification of a group of plasma exosomal miRNAs that were differentially expressed in SAH patients, yielding six miRNAs that were significantly differentially expressed in SAH patients and control plasma exosome samples: hsa-miR-369-3p, hsa-miR-136b-3p, hsa-miR-410-3p, hsa-miR-195-5p, hsa-miR-486-3p, and hsa-miR-193b-3p (Table 2).

**Table 2:** Selected differentially expressed miRNAs in NGS

miRNA name	log2 (fold change)	*P* value	Expression in SAH (vs control)
hsa-miR-369-3p	− 1.17	0.036	Downregulated
hsa-miR-136-3p	− 1.06	0.042	Downregulated
hsa-miR-410-3p	− 1.55	0.0079	Downregulated
hsa-miR-195-5p	1.03	0.027	Upregulated
hsa-miR-486-3p	1.03	0.018	Upregulated
hsa-miR-193b-3p	1.08	0.018	Upregulated

### Validation of NGS data by qRT-PCR in SAH patient cohort and SAH model in mice

**Fig. 1:**
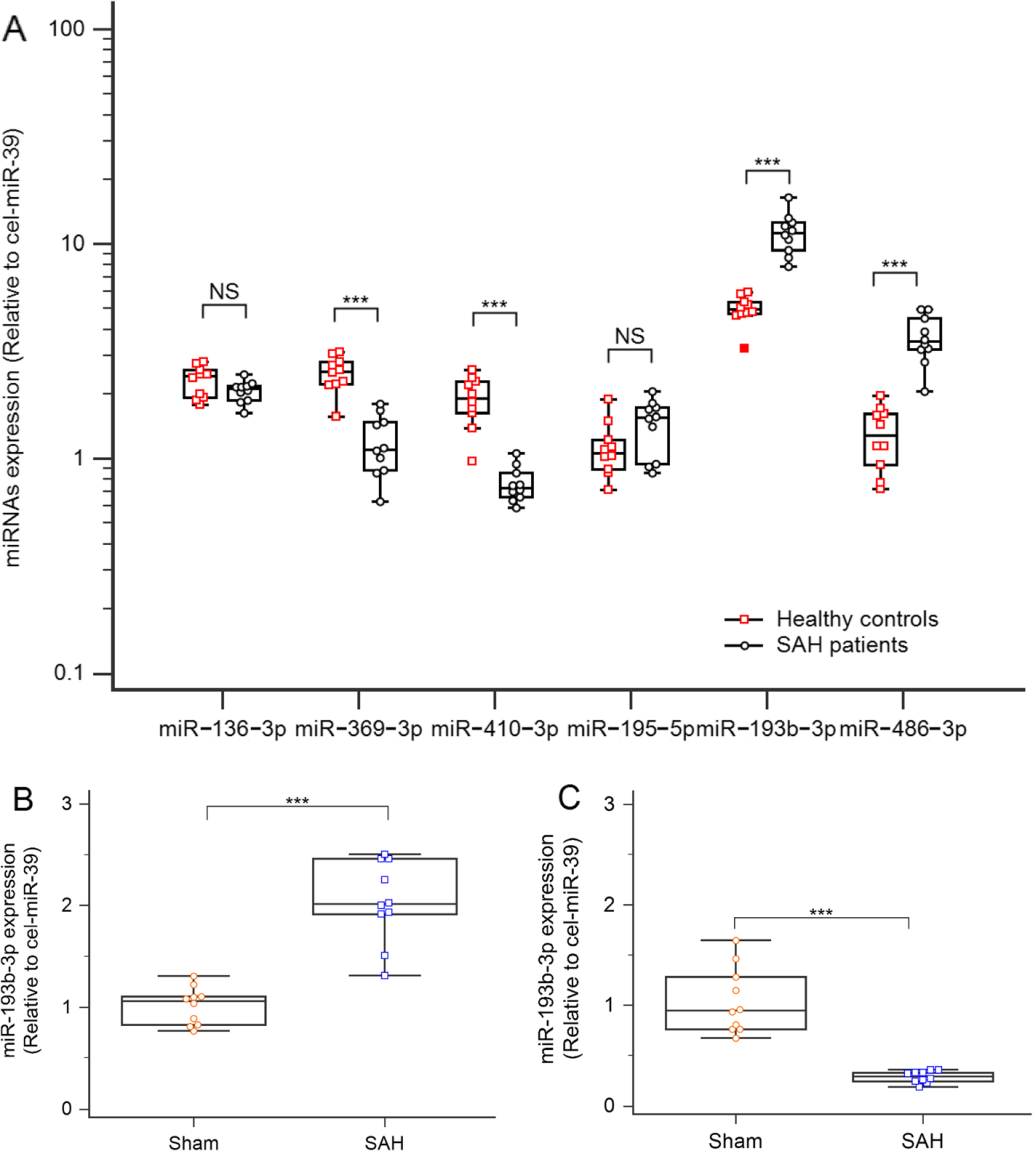
Expression levels of circulating exosomal miRNAs and miRNA in the brain after SAH. **a** Expression profiles of exosomal miRNAs after SAH. **b** Expression of miR-193b-3p in plasma exosomes of the SAH mice and sham mice. **c** Expression of miR-193b-3p in brains of the SAH mice and sham mice (24 h later, *n* = 10)

Expression of exosomal miRNAs from a total of 20 serum samples was analyzed, including samples from patients with SAH (*n* = 10) and samples from healthy control subjects (*n* = 10). The cohort consisted of four males and six females with a median age of 60 years (age range 55–67 years). Healthy controls consisted of four males and six females with a median age of 61 years (age range 54–66 years). We determined whether these six plasma exosomal miRNAs could serve as circulating markers by comparing their plasma levels between SAH patients and normal controls. qRT-PCR confirmed that four miRNAs (hsa-miR-369-3p, hsa-miR-410-3p, hsa-miR-193b-3p, and hsa-miR-486-3p) showed significantly differential expression between the experimental group (24 h post-SAH) and healthy controls (Fig. 1a). To further confirm the significance of the observed differences in exosomal miR-193b-3p expression, the expression of plasma exosomal miR-193b-3p remained statistically significant relative to controls in a mice SAH model, whereas expression levels of miR-193b-3p were lower than controls in brain tissues of SAH mice (Fig. 1b, c).

### Manufacture and characterization of RVG/Lamp2b-modified exosomes

**Fig. 2:**
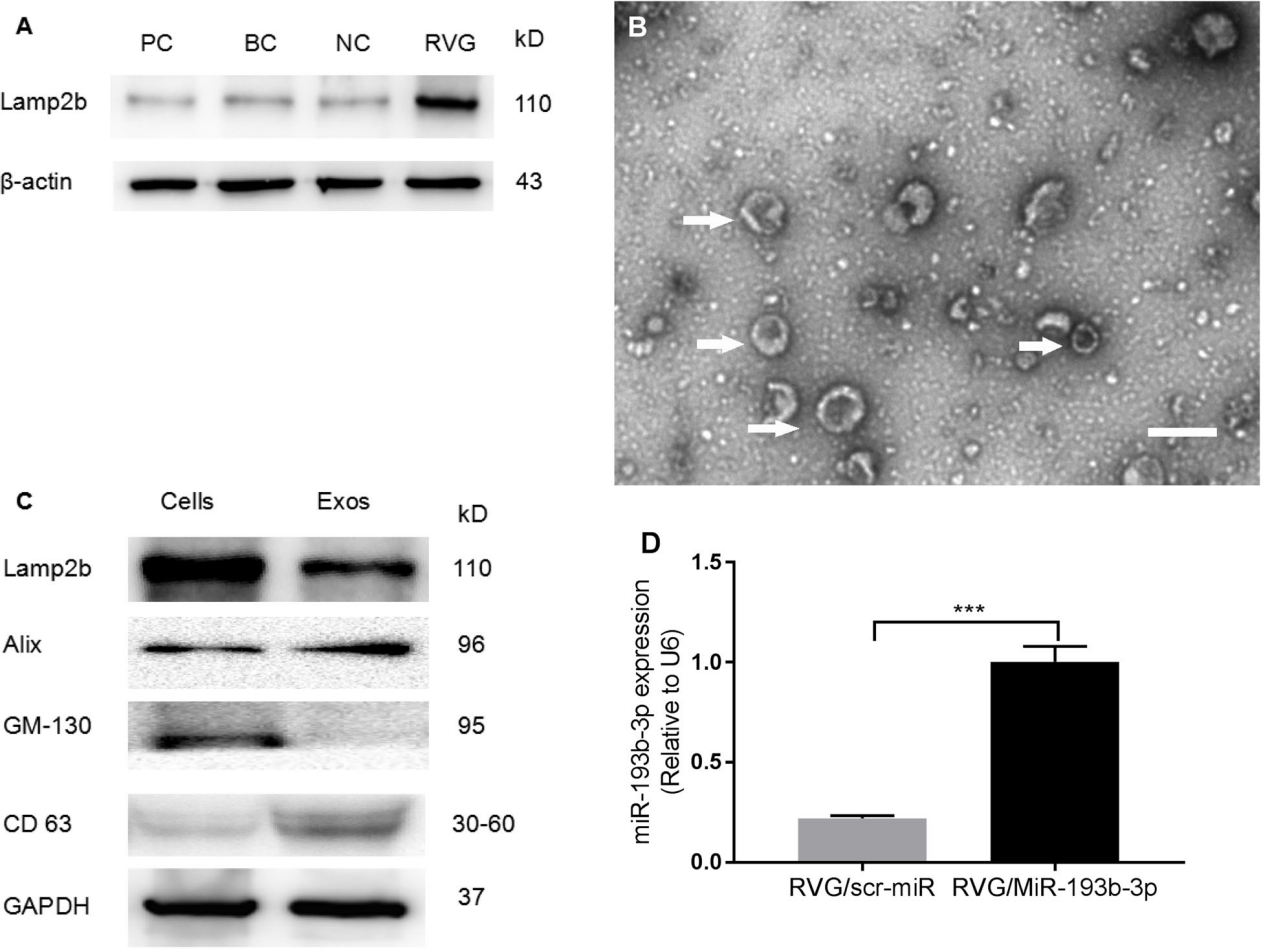
Characterization of exosomes. **a** Lamp2b in transfected BMSCs and controls (PC: positive control, HepG2; BC: blank control, BMSCs; NC: normal control, BMMSCs) were exposed to control lentivirus-infected. **b** EM image of modified exosomes derived from BMSCs. Scale bars = 200 nm. **c** Western blot analysis of Lamp2b, CD63, GM130, and Alix in RVG-Lamp2b-modified exosomes and parental cells. **d** Expression levels of miR-193b-3p quantified in exosomes in RVG/Exos/scrambled miRNAs (Control) vs RVG/Exos/miR-193b-3p using qRT-PCR analysis. The data were normalized to U6 expression and expressed as mean ± SD (***p < 0.001)

To generate RVG/Lamp2b/Exos, BMSCs were transduced with lentiviral vectors of HBLV-RVG/Lamp2b plasmid or negative control (NC). We used western blot analysis to measure protein levels of Lamp2b in these four groups to validate whether the lentiviral vectors were successfully infected into BMSCs (Fig. 2a). Then, we purified exosomes from culture supernatants of BMSCs. To analyze the characterization of RVG/Exos derived from BMSCs, the morphology of the exosomes was observed using TEM, revealing a population with typical exosomal pellets (Fig. 2b). Western blot analysis showed that Lamp2b, CD63, and GM130 (a Golgi marker), as well as the endocytic pathway and formation-associated proteins Alix were expressed in purified exosomes (Fig. 2c). Next, we examined the efficacy of exosome-encapsulated cargoes. MiR-193b-3p levels in isolated exosomes that were loaded with miR-193b-3p mimics or scrambled miRNAs via electroporation were measured using qRT-PCR analysis. We found that levels of miR-193b-3p in exosomes transfection with miR-193b-3p mimics were significantly higher than in those transfected with the negative control (Fig. 2d).

### Engraftment of targeted RVG/Exos in the brain

**Fig. 3:**
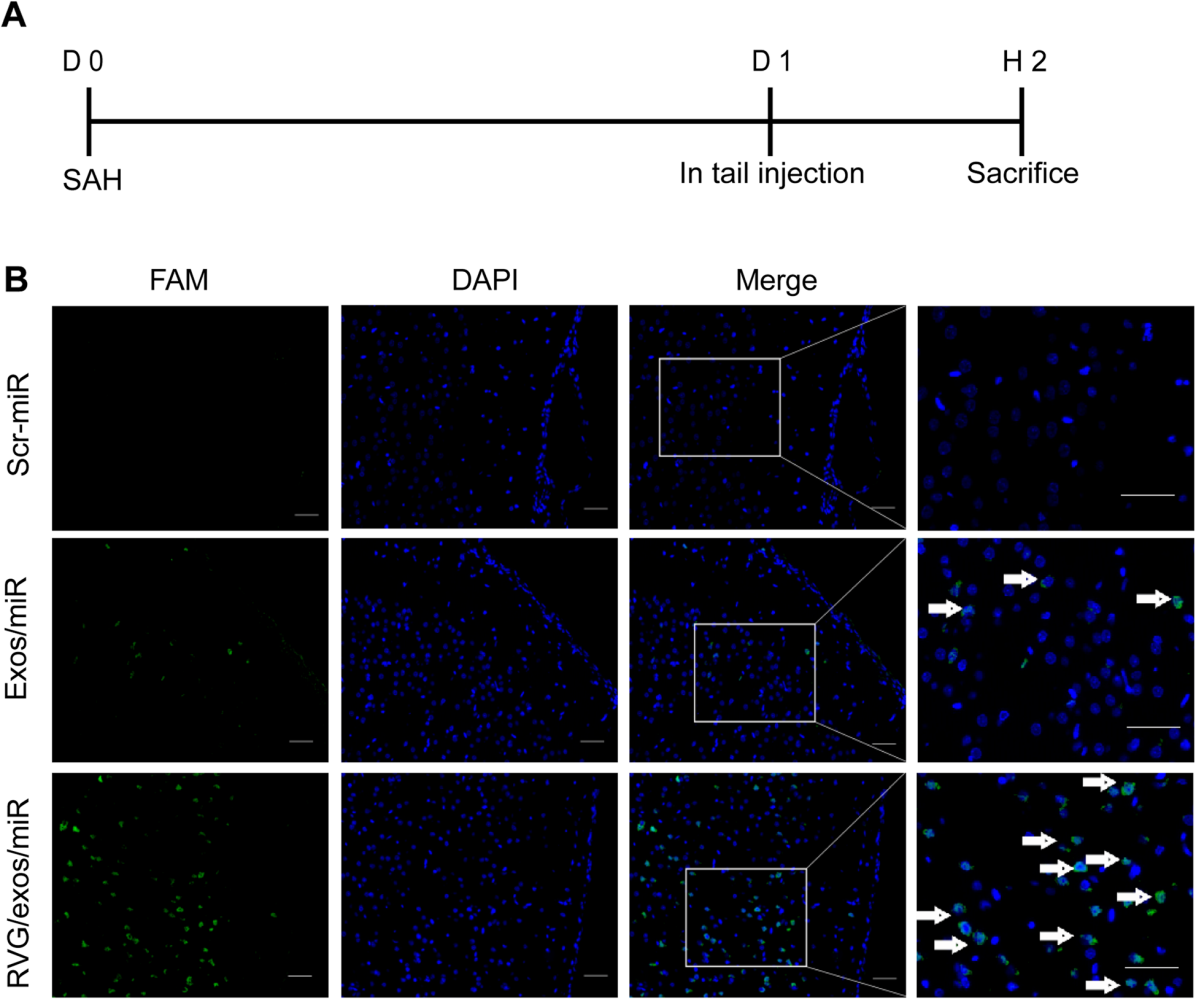
Enhanced engraftment of exosomes in brain tissues of mice. **a** Strategy of establishing the SAH model and FAM labeling, mice were sacrificed 2 h (H2) after injection. **b** Immunofluorescence images of brain tissues of mice after SAH receiving free FAM-labeled scrambled miRNAs, and unmodified Exos and RVG/Exos electroporated with free FAM-labeled scrambled miRNAs. The right panel (only last column) with boxes represented a higher magnification images, and the other images represented a lower magnification images from the boxes on the left column. Scale bars = 50 μm

To detect the presence of the RVG/Exos in the brain, we established a SAH model in mice. Unmodified/Exos and RVG/Exos were loaded with FAM-labeled miR-193b-3p. The slides from brains in the SAH model were observed at 2 h after injection (Fig. 3a). We found substantially more FAM-labeled Exos in the inferior basal temporal lobe of mice in the RVG/Exos group than that in the unmodified/Exos group, and almost no FAM could be observed in the inferior basal temporal lobe of the miR-193b-3p group (Fig. 3b). These data suggested that, compared with unmodified/Exos, RVG/Exos efficiently delivers miR-193b-3p into the hemorrhage region of the brain. MiRNAs were barely endocytosed by the cells independently. Another interesting finding was that FAM was localized mainly in the nucleus. These results suggested that miR-193b-3p target genes were probably localized in the nucleus (Fig. 3b).

### Expression patterns of histone deacetylase 3 in SAH and effects of miR-193b-3p on HDAC3 target expression

**Fig. 4:**
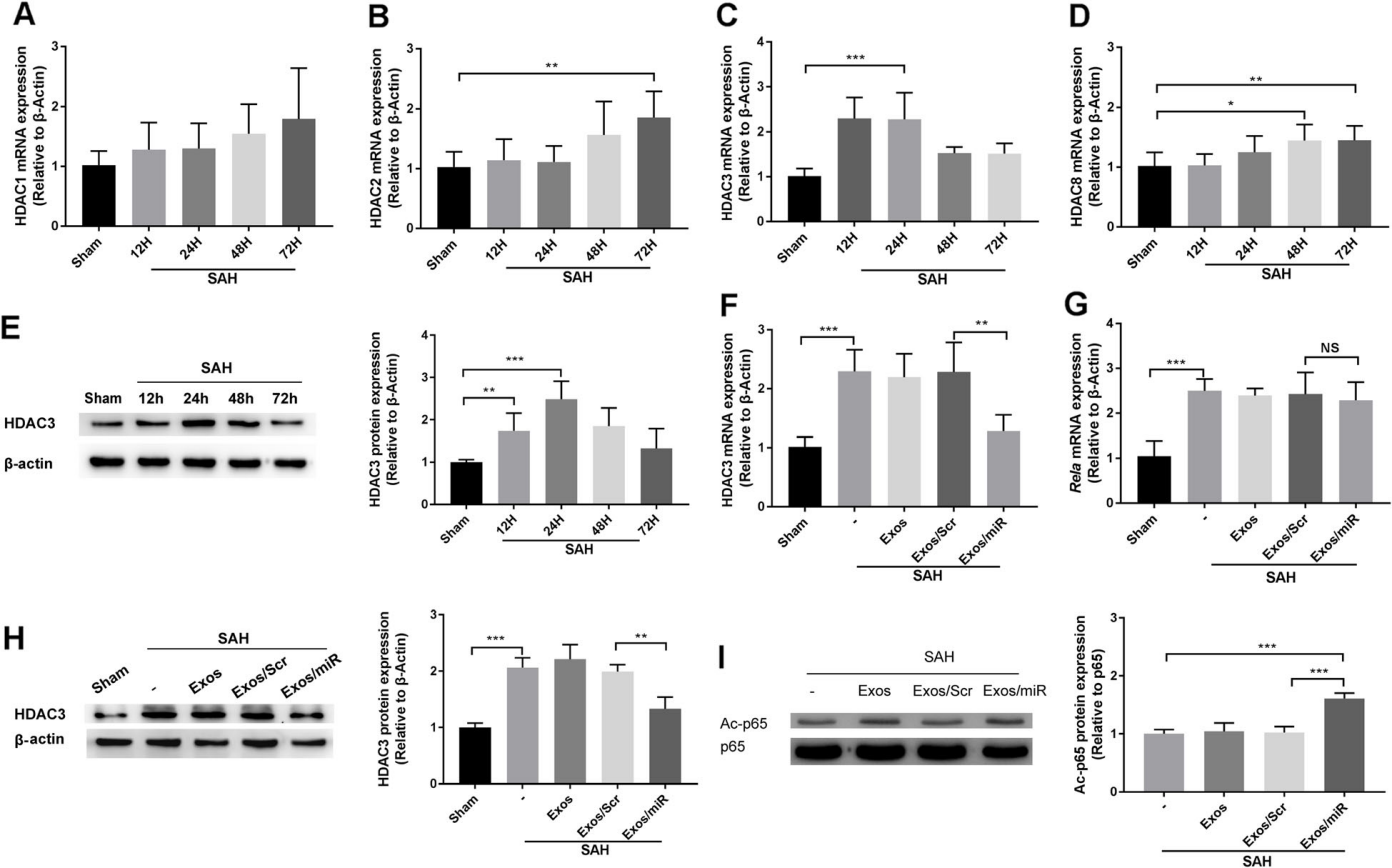
Effects of exo/miR-193b-3p on putative mRNA target expression. **a**–**d** qRT-PCR analysis and western blot analysis showed the expression levels of class I HDACs (*Hdacs* 1, 2, 3, 8) at 12, 24, 48, and 72 h after SAH (*n* = 6). **e** Western blot analysis showed the expression levels of HDAC3 at 12, 24, 48, and 72 h after SAH (*n* = 6). **f**, **g** Changes in expression of *Hdac3* and *Rela* mRNA in the brain tissues of Exos/miR-193b-3p- or Exos/Scr-treated mice were assessed (*n* = 6). **h** Expression levels of HDAC3 as well as quantifications in each group. **i** Western blot analysis of the protein level of ac-p65 in nuclear protein. The data were normalized to β-actin or p65 expression and were expressed as mean ± SD (*p < 0.05, **p < 0.01, and ***p < 0.001)

Using miRNA target prediction algorithms (TargetScan 7.0 and miRanda 3.3a), we identified the potential targets of miR-193b-3p. We also noticed that miR-193b-3p has the potential to regulate HDAC3 expression in a previous study [29]. HDACs in human are divided into four classes. Class I (HDACs 1, 2, 3, 8) is localized mainly in the nucleus and expressed in all tissues; their highest expression is observed in the brain [30, 31]. We then measured the dynamic changes and expression levels of class I HDACs in the brain of mice after SAH. We found that, in comparison with the sham group, the mRNA levels of *Hdac3* reached peak levels at 12–24 h after SAH, and protein levels of HDAC3 reached a peak at 24 h after SAH (Fig. 4c, e). We also found that, in comparison with the sham group, expression levels of class I HDACs (HDACs 1, 2, 8) did not reach peak levels before 24 h after SAH (Fig. 4a, b, d). Lysine acetylation of the NF-κB p65 (*Rela*) has been shown to be indispensable for the NF-κB pathway activity regarding inflammatory mediators, playing a vital role in response to damage stimuli [32, 33]. We found that NF-κB p65 expression levels were higher in the SAH group than in the sham group (*P* < 0.001). *Rela* mRNA levels were not significantly different after the Exos/miR-193b-3p treatment (Fig. 4g). HDAC3 levels were lower after Exos/miR-193b-3p treatment (Fig. 4f, h), whereas ac-p65 protein levels were higher in the nucleus (Fig. 4i). Our data suggest that miR-193b-3p suppresses HDAC3 expression and activity, leading to increased ac-p65 protein levels.

### Expression levels of *BCL-2*, *caspase-3*, IL-1β, IL-6, and TNF-α in various groups

**Fig. 5:**
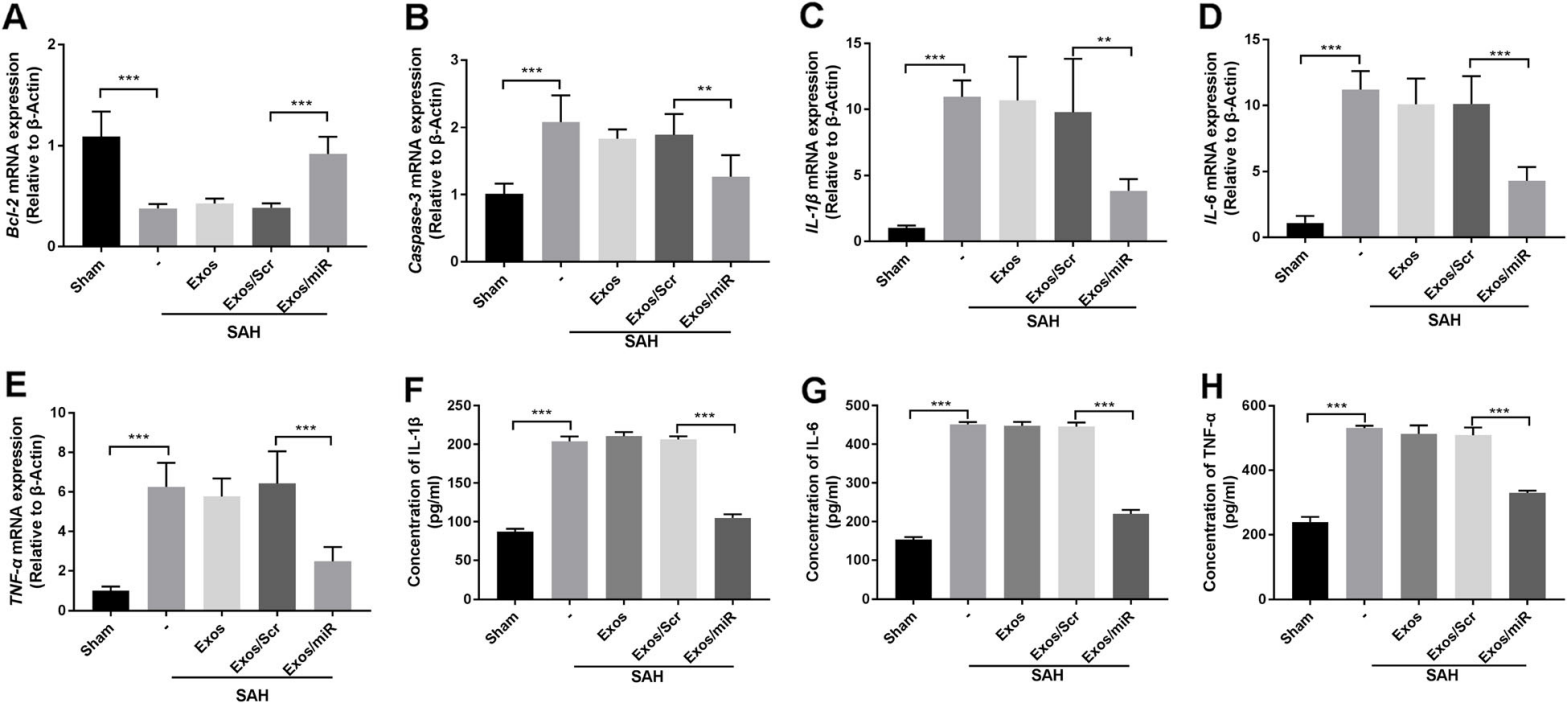
Exo/miR-193b-3p treatment suppressed apoptosis parameters and decreased levels of the inflammatory cytokines IL-1β, IL-6, and TNF-α after SAH. **a**–**e** Changes in expression of *Bcl-2*, *caspase-3*, *IL-1β*, *IL-6*, and *TNF-α* in the SAH model as assessed by qRT-PCR. The data were normalized to β-actin expression and expressed as mean ± SD (**p < 0.01, ***p < 0.001, and NS: not significant; *n* = 6). **f**–**h** The results of ELISA for levels of the inflammatory factors IL-1β, IL-6, and TNF-α in mouse brain tissues, *n* = 6 (***p < 0.001)

We used qRT-PCR to quantify downstream gene expression levels in the NF-κB pathway. *Caspase-3* gene expression was elevated after SAH and lower after Exos/miR-193b-3p treatment (Fig. 5a). Interestingly, we found that *Bcl-2* expression was lower after SAH and was dramatically greater after Exos/miR-193b-3p treatment (Fig. 5b). The mRNA levels of *IL-1β*, *IL-6*, and *TNF-α* were higher after SAH, and Exos/miR-193b-3p significantly reduced the expression of proinflammatory cytokines (Fig. 5c–e). Levels of IL-1β, IL-6, and TNF-α in the cerebral hemisphere were measured using ELISA. We found that inflammatory cytokine levels were substantially higher with biphasic peaks after SAH. The data suggested that Exos/miR-193b-3p significantly lowered expression levels of IL-1β, IL-6, and TNF-α in the brain after SAH (Fig. 5f–h).

### The effect of miR-193b-3p on neurological behavioral impairment, brain edema, neurodegeneration, and BBB permeability in mice after SAH

**Fig. 6:**
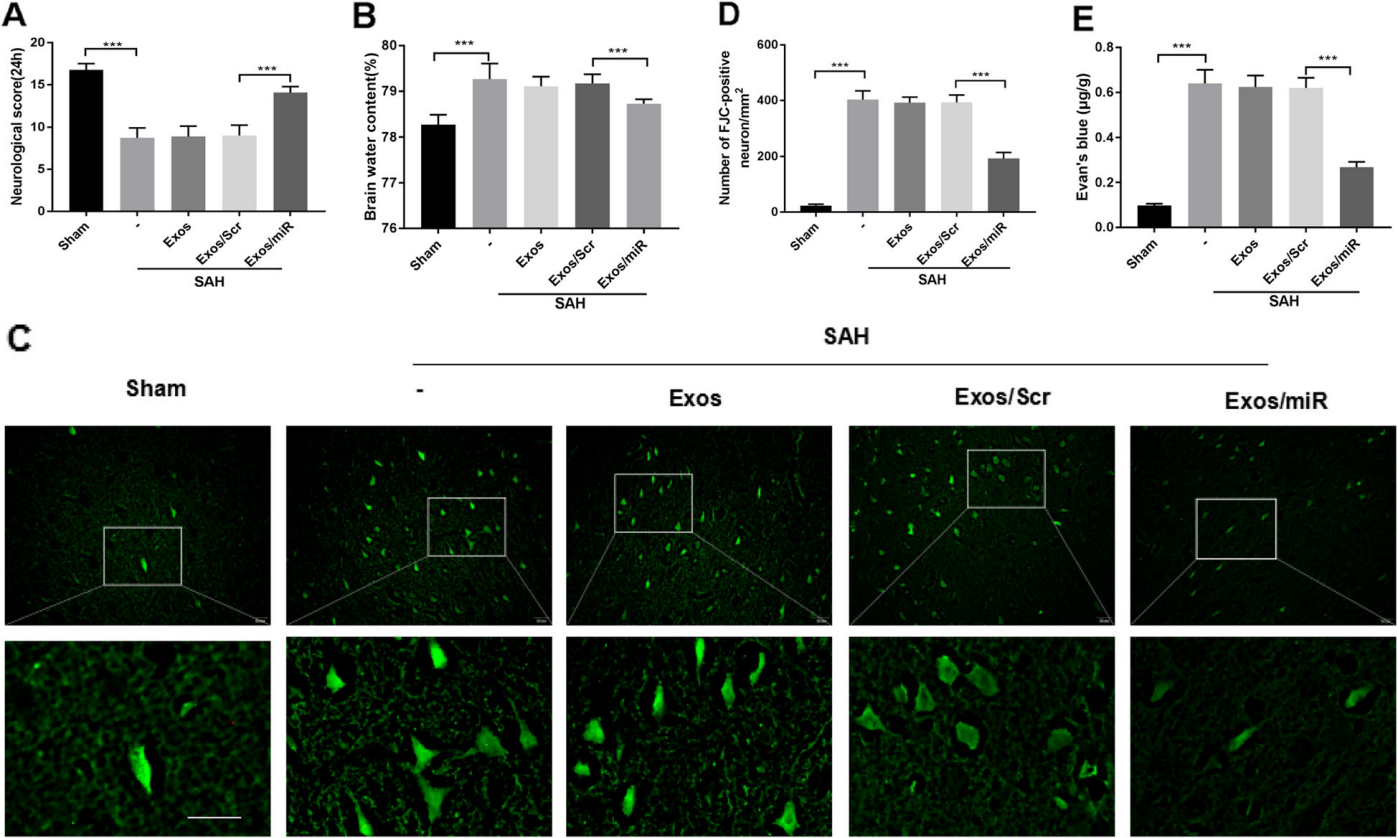
Effects of Exo/miR-193b-3p treatment on neurological behavioral impairment, brain edema, BBB injury, and neurodegeneration in SAH mice. **a** Evaluation of neurological scores in each group (*n* = 18). **b** Alterations in brain water content in each group (*n* = 6). **c**, **d** FJC staining and the number of FJC-positive cells in every square millimeter of brain tissue. The upper panel (only first row) with boxes represented lower magnification images, and the other images represented higher magnification images from the boxes in the first row. Scale bars = 20 μm. **e** Quantitative analyses of Evan’s blue dye extraversion (*n* = 6). All quantitative data are presented as mean ± SD (*p < 0.05, **p < 0.01, ***p < 0.001, and NS: not significant)

To determine the protective function of miR-193b-3p at 24 h post-SAH, neurological behavioral impairment, neurodegeneration, BBB permeability, and brain water content of mice in various groups were tested. As shown in Fig. 6a, the SAH group showed lower neurological scores than the sham group (*p* < 0.01). Exos/miR-193b-3p markedly improved neurological scores compared with the SAH and Exos/Scramble miRNA groups at 24 h post-SAH. Similarly, the degree of brain edema was significantly less pronounced in the Exos/miR-193b-3p group than in the SAH and Exos/Scramble miRNA groups (Fig. 6b). The numbers of FJC-positive cells were lower in the Exos/miR-193b-3p group and were higher in the SAH and Exos/Scramble miRNA groups compared with the sham group (Fig. 6c, d). SAH produced the amount of Evan’s blue extravasation into the brain, which revealed the BBB injury (*p* < 0.001, Fig. 6e). Also, enhanced Evan’s blue extravasation was found in the Exos group and Exos/Scramble miRNA group as compared with the sham group. However, the BBB permeability was significantly attenuated by the Exos/miR-193b-3p treatment (*p* < 0.001, Fig. 6e).

## Discussion

In the present study, firstly, we found that the circulating exosomal miRNA expression profiles showed distinct pattern differences between the SAH patients and healthy controls. Secondly, a SAH model of mice showed that plasma exosomal miR-193b-3p was greater than that in the sham controls, while it has lower expression in brain tissues. Thirdly, RVG-modified exosomes deliver miRNAs into the central nervous system (CNS) [11, 34], suggesting that miR-193b-3p delivery may be achieved using exosome encapsulation. Finally, we found that targeted delivery of miR-193b-3p into the brain after SAH reduced neuroinflammation and attenuated neuronal degeneration by inhibition of the HDAC3/NF-κB signal pathway.

We started with a total exosomal miRNA analysis using NGS to identify changes in expression levels of miRNAs between SAH patients and healthy controls. Subsequently, the NGS results for hsa-miR-486-3p, hsa-miR-193b-3p, hsa-miR-369-3p, and hsa-miR-410-3p were verified using qRT-PCR experiments in a training set. We selected miR-193b-3p for follow-up validation to identify its role in a SAH mouse model. Exosomes can be enriched from peripheral blood samples released by brain cells and endothelial and peripheral cells. Exosomal miRNAs also can transport epigenetic information, affecting gene expression in cells distant to the cellular source [22, 35]. Exosomes are a newly described mechanism of intercellular communication facilitating transfer of miRNAs, lipids, mRNAs, and proteins [36]. Importantly, exosome delivers miRNA or protein to the brain [25, 37]. Furthermore, modified exosomes with RVG fused to membrane glycoprotein Lamp2b efficiently delivered miRNA to the brain [34]. In our study, we found that FAM fluorescence was ferried to the brain of mice by exosomes. Originally, intravenously injection of DiI fluorescence ferried by RVG-Exos was used to achieve tailored delivery [11, 25, 34, 38]. RVG-exosomes delivered exogenous miR-124 to ischemic cortex and attenuated ischemic injury via cortical neurogenesis [11]. RVG-modified MSC-exosomes effectively balanced the inflammatory responses and improved learning and memory function in the brains of mice with Alzheimer’s disease [25]. RVG-exosomes delivered α-Syn siRNA to reduce alpha-synuclein aggregates in brains of mice with Parkinson’s disease [38]. In this study, we also confirmed that RVG/Exos could successfully deliver miR-193b-3p to the brain using loaded exosomes with FAM-labeled scrambled miRNAs.

The inflammatory response occurred early after SAH and contributes to the progression of SAH-induced EBI [5, 6]. Therapeutically, inhibition of neuroinflammation would be greatly beneficial to alleviate inflammation-induced EBI after SAH. Using this RVG/Exos/miR-193b-3p in vivo treatment strategy, our data suggested that intravenous injection of miR-193b-3p loaded RVG/Exos causes significant decreases in HDAC3 mRNA and protein levels in brain tissues. Previous studies showed that HDAC3 was a class I histone deacetylase [39]. Histone deacetylase inhibition has a neuro-protective effect via reducing neuroinflammation [33], reported to be beneficial with respect to neurological functions in many diseases, including Alzheimer’s disease [40], Huntington’s disease [41], TBI, stroke [42], and spinal cord injury [33]. Recent studies reported that HDCA3 inhibition appears to suppress NF-κB transcriptional activity by maintaining the NF-κB p65 acetylated (inactive) state and restraining the inflammatory response [33, 43]. The NF-κB signaling pathway occurs in passive post-injury necrosis and in damaged cells, activating microglia to secrete inflammatory cytokines and causing amplification of the inflammatory response cascade [44, 45]. In the present study, we found that levels of inflammatory cytokines (IL-1β, IL-6, and TNF-α) in brain tissue increased after SAH, associated with apoptosis (caspase-3, BCL-2), brain edema, and neurological defects. Inhibition of HDAC3 activity following miR-193b-3p treatment reduced expression levels of these inflammatory cytokines in vivo. NF-κB should be a transcription factor of inflammatory mediators that plays a key role in neuroinflammation [46, 47]. MiR-193b-3p significantly increased NF-κB p65 acetylation and reduced its transcriptional activity after SAH, suggesting that miR-193b-3p exerts neuroprotective effects. A previous study reported that miRNA-193b-3p inhibited hepatocyte apoptosis in selenium-deficient chickens [48]. Another study showed that HDAC3 deletion restrained neuroinflammation by regulating NF-κB p65 signaling, leading to neuroprotective effects after spinal cord injury. Previous studies also showed that HDAC3 inhibition had neuroprotective effects in stroke. HDAC3 inhibition increased the resistance of the brain to ischemic insult following middle cerebral artery occlusion (MCAO) [49]. HDAC3 inhibition exerted protective effects against diabetic stroke via the enhancement of oxidative stress, inhibition of apoptosis, and improvement of autophagy, all of which might be mediated by the upregulation of Bmal1 [50]. Furthermore, miR-494 in MCAO mouse model produced neuroprotective effects by reducing the expression levels of HDAC3 in neurons [51].

Although the present study aimed at investigating the tailored delivery of RVG-exosomes and potential mechanism of miR-193b-3p in neuroinflammation after SAH, there were a few methodological limitations. First, HDAC3 was a potential target of miR-193b-3p in brain tissues of mice after SAH, but it is not the only one. Second, miR-193b-3p attenuated neuroinflammation and led to neuroprotective effects after SAH. HDAC3 inhibitors acted on several cell types in the CNS, including neurons, astrocytes, and microglia [52]. miR-193b-3p and its target HDAC3 may also be regulated by other mechanisms, including autophagy or apoptosis [49, 51], possibly leading to neuroprotective effects. Further studies are required to account for the beneficial mechanisms of miR-193b-3p in vitro.

## Conclusions

Taken together, our data suggested that intravenous injection of RVG-exosomes containing miR-193b-3p in a SAH mouse model promotes neuroprotective effects and anti-inflammatory responses. Inhibition of inflammation by miR-193b-3p affects the acetylation status of NF-κB p65 and reduces the expression of HDAC3. These findings suggest that RVG/Exos/miR-193b-3p may weaken neuroinflammation by suppressing the expression and the activity of HDAC3 and increasing NF-κB p65 acetylation after SAH.

## Supplementary information

**Additional file 1.**

**Additional file 2.**

## Data Availability

The datasets used and/or analyzed during the current study are available from the corresponding author on reasonable request.

## Abbreviations

Ac-NF-κB: Acetylation of nuclear factor-κB
BBB: Blood-brain barrier
BCA: Bicinchoninic acid
BCL-2: B cell lymphoma-2
BMSCs: Bone marrow mesenchymal stem cells
BWC: Brain water content
CNS: Central nervous system
DCI: Delayed cerebral ischemia
DW: Dry weight
EBI: Early brain injury
ECL: Enhanced chemiluminescence
ELISA: Enzyme-linked immunosorbent assay
Exos: Exosomes
FJC: Fluoro-Jade C
HDAC3: Histone deacetylase 3
Lamp2b: Lysosome-associated membrane glycoprotein 2b
MiRNAs: MicroRNAs
NF-κB: Nuclear factor-κB
NGS: Next-generation sequencing
PBS: Phosphate buffer solution
PVDF: Polyvinylidene difluoride membranes
qRT-PCR: Quantitative real-time polymerase chain reaction
RVG: Rabies virus glycoprotein
SAH: Subarachnoid hemorrhage
SDS-PAGE: Sodium dodecyl sulfate-polyacrylamide gels
TNF: Tumor necrosis factor
WW: Wet weight
